# Evaluation of Immunoglobulin G Absorption from Goat Colostrum by Newborn Piglets

**DOI:** 10.3390/ani10040637

**Published:** 2020-04-07

**Authors:** Silvia Martínez Miró, Susana Naranjo, Josefa Madrid, Miguel José López, Cristian Jesús Sánchez, Mónica Marcela Segura, Fuensanta Hernández

**Affiliations:** Department of Animal Production, Faculty of Veterinary, University of Murcia, Campus de Espinardo, 30100 Murcia, Spain; susinaranjo22@gmail.com (S.N.); alimen@um.es (J.M.); mjlopeza@um.es (M.J.L.); cristianjesus.sanchez@um.es (C.J.S.); monicamarcela.segurar@um.es (M.M.S.); nutria@um.es (F.H.)

**Keywords:** goat colostrum, immunoglobulin, neonatal piglet

## Abstract

**Simple Summary:**

Alternatives to sow colostrum are necessary to ensure adequate colostrum intake by piglets born from hyperprolific sows. This study was conducted to evaluate whether piglets can absorb goat immunoglobin G (IgG) and study its effects on piglets. The results showed that piglets absorbed goat IgG with an apparent coefficient of absorption of 20.9%. In addition, the piglets tolerated goat colostrum well, opening up the possibility of developing supplements based on goat colostrum for newborn piglets.

**Abstract:**

The aim of this study was to evaluate whether piglets absorb immunoglobin G (IgG) from goat colostrum and the potential effects of its ingestion on suckling piglets. Thirty-eight piglets with body weights ranging from 1000 to 1700 g were assigned to one of the three experimental treatments: Control group (C), where piglets were allowed to suckle normally, and porcine and goat groups. The piglets from the last two groups were removed from the sows after birth and received an oral 20 mL dose every 3 h of porcine (PC) or goat colostrum (GC), respectively, during first 12 h of life. Then, they were returned to newly farrowing sows to continue suckling until 20 d. The apparent efficiency of absorption (AEA) of IgG at 12 h was calculated as total serum IgG divided by ingested IgG. No diarrhea or symptoms of intolerance were observed at any time. On day 20, body weight and the number of dead piglets were similar in all three treatments (*p* > 0.05). At 12 h, the concentration of goat IgG in the serum of piglets fed GC was 8.11 mg/mL. AEA was 20.9% for goat IgG and 26.3% for porcine IgG (*p* > 0.05). Therefore, goat colostrum seems a promising alternative to study new feed supplements or artificial rearing of newborn piglets.

## 1. Introduction

Adequate colostrum intake by neonatal piglets is crucial for their energy and immunoglobulin supply since piglets are born with low energy reserves and immunoglobulin transfer via placenta is negligible due to the epitheliochorial nature of the porcine placenta [1]. Immunoglobulin concentration in colostrum is highest during the birth process but declines rapidly in first 24 h of secretion. Moreover, the transfer of intact macromolecules across the gastrointestinal tract is only possible for a short time after birth [2], so the rapid ingestion of colostrum after birth is decisive for the survival of piglets. In addition, colostrum is an important source of bioactive substances and hormones associated with the development of newborns [2,3].

An adequate intake and absorption of colostrum may be difficult for piglets due to many factors, among which are birth order, low birth weight, and piglet vitality [4]. The breeding of hyperprolific sows with an increase in average litter size has further increased the risk of newborn piglets dying from starvation and lack of passive immunity [1,5]. Since colostrum production is highly variable among sows and independent of litter size [6], ensuring a sufficient colostrum uptake by all piglets is an important challenge. For this purpose, immunoglobulins and energetic supplements have been provided from many different sources (colostrum, plasma, serum, slaughterhouse blood, commercial products) [7].

Supplements of porcine immunoglobulins have been used to improve the growth and survival rates of colostrum-deprived pigs. Bikker et al. [8] and Campbell et al. [9] showed that newborn piglets absorbed immunoglobin G (IgG) from oral supplements containing IgG from porcine plasma and energy substrate, improving the viability and immune competency of neonatal piglets. Furthermore, immunoglobulins from bovine sources have been used to provide passive immunity in piglets. Drew and Owen [10] showed that bovine serum immunoglobulins mixed with sow replacer milk were well tolerated by the piglets, although the porcine immunoglobulins were better absorbed than the bovine. Gomez et al. [11] indicated that both porcine immunoglobulins and bovine colostrum could be used as immunoglobulin sources in artificial pig rearing. Similarly, Jensen et al. [5] concluded that bovine colostrum mixed with porcine plasma may be a useful substitute for porcine colostrum in rearing of newborn pigs. However, to the best of our knowledge, no studies have been conducted using goat colostrum in newborn pigs. According to a comprehensive study, goat colostrum is a co-product of the dairy industry. It has a high nutritional value and contains immunoglobulins and other bioactive substances [12,13], which could make it a viable alternative to supplement the feeding of newborn piglets. In addition, fat globules are smaller in goat than cow milk, which makes it easily digestible. Moreover, digestibility of goat milk protein is higher compared to cow milk protein and, in general, goat milk has a greater potential than other animals’ milk to prevent various diseases [14,15].

We hypothesized that if piglets tolerated bovine colostrum and were able to absorb bovine IgG, goat colostrum and IgG could be absorbed and tolerated in a similar pattern to bovine colostrum by piglets. The present study aimed to evaluate whether piglets absorb IgG from goat colostrum and the potential effects of its ingestion on suckling piglets. 

## 2. Materials and Methods 

### 2.1. Ethical Approval

The experimental procedures and animal handling were in accordance with European Union guidelines for the care and use of research animals and were approved by the Ethics Committee of Animal Experimentation of the University of Murcia and the Administrative Authorities (A-13170805).

### 2.2. Animals and Management

The study was conducted on a commercial farm located in Huércal-Overa (Almería, Southeastern Spain) with 4000 Large White x Landrace sows. The sows were inseminated with Pietrain sperm and housed in individual pens (2.5 m long × 0.63 m wide) until gestation was confirmed. Then, the sows were reallocated to pens with a minimum space of 2.5 m^2^ per animal, in compliance with the European Union regulations concerning the protection of animals used for experimental and other scientific purposes (EU Directive 2010/63/EU). Five days before farrowing, the sows were moved to farrowing house and placed in individual crates (2.36 m long × 1.5 m wide), where they stayed until the end of lactation at 21 days. The piglet area was heated by under-floor heating. During pregnancy, sows were fed 2.5 kg of commercial pregnant diet containing 12.2 MJ/kg metabolizable energy (ME), 13 mg/kg crude protein, and 0.6 mg/kg lysine. After delivery, sows were initially fed 2.5 kg of commercial lactating diet containing 12.9 MJ ME/kg, 16 mg/kg crude protein, and 0.8 mg/kg lysine. This amount was increased daily by 0.5 kg until the maximum quantity was reached. Feed composition and sow management for each state followed the recommendations of the Spanish Foundation for the Development of Animal Nutrition (Fundación Española para el Desarrollo y Nutrición Animal, FEDNA) [16]. Water was provided ad libitum. The ambient temperature in the farrowing unit was kept at approximately 20 °C and ventilation was thermostatically controlled.

Twenty multiparous sows (parity ranged from 2 to 6) housed in the same farrowing room and with similar expected farrowing date were selected for this trial. Only sows with 3 or 6 valid piglets in their litter (body weight at birth between 1000–1700 g and with no obvious defects) were used, because 3 piglets (one per treatment) were selected from the same sow, with up to a maximum of 6 piglets selected from the same sow. Finally, seven sows were used. 

Thirty-eight piglets from Pietrain × (Large White × Landrace) with body weights between 1000 and 1700 g and with no obvious defects were used. Immediately after birth, the piglets were dried, weighed, and individually identified by an ear tag before being assigned to one of the three experimental treatments: Control group (C) (n = 13), piglets receiving pig colostrum (PC) (n = 13), and those receiving goat colostrum (GC) (n = 12). Piglets in group C were kept with their own sows and allowed to suckle as normal. Piglets in the PC and GC groups were removed from the sows immediately after birth and placed in plastic containers with the temperature maintained at 30–32 °C using a heating lamp during the first 12 h after birth.

The piglets within the PG and GC groups received an oral 20 mL dose every 3 h of sow or goat colostrum, respectively, starting at 0 h after birth (time 0) and ending at 12 h, a total of 5 doses per piglet, following a protocol similar to that used by Jensen et al. [5]. The feed was heated to 35 °C before feeding. After the last dose, they were returned to a newly farrowing sow to ensure natural suckling. Subsequently, all piglets followed the same management practices of the farm for the 20 days that the study lasted. 

For manual colostrum administration, piglets were held from behind the head with thumb and index finger extending to the corners of the piglet’s mouth. Gentle pressure was applied to open the piglet’s mouth and a bottle with colostrum was inserted. When the piglet had ingested the entire dose, it was gently released into the container. 

### 2.3. Measurements 

All experimental piglets were handled similarly. Due to the importance of thermoregulation of piglets immediately after birth, it was necessary to know if artificially suckling was able to maintain the piglets’ temperature. The rectal temperature of piglets was measured immediately after birth and 24 h later using a digital thermometer (Thermometer für Großtiere SC 212, SCALA Electronic GmbH, Germany). Body weight was measured in piglets with an electronic scale at birth, at 12 h and 24 h postpartum, and then on day 10 and 20 after birth.

### 2.4. Tolerance of Goat Colostrum 

Any incidence of disease, diarrhea, or death was recorded daily throughout the experiment (1–20 d). The feces score was estimated on the scale previously described by Gomez et al. [11]: 1 = normal, solid feces, 2 = soft, looser than normal stools, 3 = liquid diarrheal feces. All observations were made by the same observer. In order to establish the cause of death, a postmortem examination was conducted on any piglets that died during the assay.

### 2.5. Samples Collection

Blood samples (2 mL) from each piglet were collected by jugular venipuncture using hypodermic needle (0.6 × 25 mm, 23G) and syringe. Once collected, blood samples were transferred into vacuum tubes without additives (Vacuette®, Greiner Bio-One, Kremsmünster, Austria). The samples were stored at 4 °C and transported to the laboratory within 1 h after collection and then they were centrifuged at 2000× *g* for 10 min. The serum was immediately frozen at −20 °C until further analysis. The blood samples were obtained at 12 h and on 10 and 20 d and were used to quantify the concentration of serum IgG. 

### 2.6. Colostrum Collection

Three weeks prior to this study, porcine colostrum was collected manually from a total of seventeen multiparous sows (Large White × Landrace) within 3 h of starting farrowing. The colostrum was pooled, pasteurized at 55 °C for 80 min, and stored frozen at −20 °C. Goat colostrum was obtained from the first milking of the first postpartum day of fifty dairy multiparous goats by mechanical milking on a commercial farm. Colostrum was stored at −20 °C after pasteurizing at 55 °C for 80 min. Samples of each pool of colostrum were collected to analyze the chemical composition by infrared spectrophotometry (MilkoScan FT120; Foss Electric A/S, Hillerød, Denmark; IDF, 2000), and the immunoglobin G (IgG) level was determined using ELISA kits.

### 2.7. Quantification of Immunoglobulins

Assays for pig and goat IgG were performed using specific ELISA quantification kits purchased from Bethyl Laboratories, Inc. (Montgomery, TX, USA). Porcine- and goat-specific immunoglobin assays were performed on colostrum samples and piglet serum as previously described by Leonard et al. [17]. The assays were performed according to the manufacturer’s instructions. The absorbance at 450 nm was measured using a microplate reader (Infinite M200PRO, Tecan Trading AG, Switzerland). The colostrum chemical composition and concentration of IgG are shown in Table 1. 

### 2.8. Absorption of Goat IgG

The apparent efficiency of absorption (AEA) of IgG at 12 h was calculated by dividing the total amount of IgG in piglets serum by the amount supplied to each piglet, taking into account that the total amount of blood in 12–24 h piglets is 10% of the body weight with a hematocrit value of 30% [18]. The following equation was used to calculate the AEA [19]:

$$AEA\;\left(\%\right)=\frac{serum\;IgG\;concentration\;\left(\frac{mg}{mL}\right)\times plasma\;volume\;\left(mL\right)}{colostral\;IgG\;concentration\;\left(\frac{mg}{mL}\right)\times intake\;colostrum\;volume\left(mL\right)}\times 100$$

The AEA was calculated for porcine and goat IgG in the serum of piglets fed PC or GC, respectively.

### 2.9. Statistical Analysis

All statistical analyses were performed using the SPSS Statistics 15.0 software (IBM SPSS, Chicago, IL, USA) with piglet as the experimental unit. Data of weight, temperature, and serum IgG concentration at each individual time-point (hour 0, 12, and 24, and day 10 and 20, in each case) were analyzed by General Linear Model with treatment as a fixed factor. The analysis was performed as a univariate analysis. Each variable was analyzed separately and predictor variable was the treatment (control, porcine colostrum, or goat colostrum). The differences among means were further compared using the Least significant difference (LSD) method. Results are given as mean values and a pooled standard error of the mean (SEM). Finally, Pearson’s Chi-Square test was used to examine relationships between mortality data at the end of the trial and the treatment, considering a significance level of *p* < 0.05.

## 3. Results

### 3.1. Growth and Rectal Temperature of Piglets

The results for body weight (BW) and weight gain are presented in Table 2. Initial and final body weights pointed to no effects of any treatment (*p* > 0.05). The piglets that received PC and GC lost significantly more body weight during first 12 h after birth (about 4%) than the piglets that remained with their own sows (C treatment), which gained weight (about 6%). However, the average weight gain did not significantly differ during at any time from 12 h to 20 days of age. 

Rectal temperature at 0 and 24 h after birth did not differ significantly among treatments (Table 2). 

### 3.2. Tolerance of Goat Colostrum: Diarrhea and Mortality

All piglets had feces score of 1, indicating that no diarrhea was observed. With regard to mortality, at 24 h, one piglet died in the PC treatment and one in the GC treatment. From 24 h to day 10, one piglet died in C and one in the PC treatment. From day 10 to 20, no piglets died in any treatment. Mortality was not significantly different between treatments (*p* > 0.05). All dead piglets were examined and crushing was diagnosed as the cause of death in all of them.

### 3.3. Immunoglobulin G

Serum concentrations of goat and pig IgG are shown in Table 3. The GC piglets showed significantly (*p* < 0.05) higher serum goat IgG levels than C and PC piglets at 12 h, while at 10 d no significant differences were observed among the different treatments and were remained at very low levels in all of them. Piglets that received PC and GC showed significantly (*p* < 0.001) lower serum pig IgG levels at 12 h and 10 d than the piglets of the C treatment. Piglets fed GC showed significantly lower pig serum IgG than the pigs of the PC treatment at both sampling times. At 20 d, pig IgG did not differ significantly among treatments (*p* > 0.05).

### 3.4. Apparent Efficiency of Absorption of Immunoglobins

The apparent efficiency of absorption of IgG at 12 h in piglets fed GC was 20.9 ± 3.24% and in piglets fed PC was 26.3 ± 2.65%. There was no effect of treatment on the AEA (*p* > 0.05).

## 4. Discussion

Adequate colostrum intake in livestock species is essential for neonatal survival. However, in pig farms breeding hyperprolific sows, the correct intake of colostrum is difficult, and several authors have studied the use of supplementation with porcine and bovine immunoglobulins [8,10,11,19,20] or energy supplements [8,9,20,21] to make up for this deficit. To date, goat colostrum for piglet feeding has not been used, so this study was conceived to evaluate whether piglets can absorb goat IgG and any effects of its ingestion. 

The chemical composition of goat and sow colostrum used in this study was in agreement with the results previously reported in the literature for goats [22] and sow [23] colostrum.

The piglets used in this study were of medium initial body weight in order to avoid using immature piglets or those of low viability and were artificially fed with 100 mL of colostrum during the 12 first hours after birth. 

Svendsen et al. [24] recommended a colostrum intake of 120 g/kg BW over the first 24 h after birth to maintain optimal levels of IgG, while Devillers et al. [25] estimated that 200 g of colostrum per piglet was the minimum consumption to provide passive immunity, allow a slight weight gain, and reduce the risk of mortality before weaning. The amount (100 mL) used in our study over the first 12 h would appear to be within the above recommendations, but, although initial body weight was similar between groups, the artificially fed piglets showed lower growth at 12 h compared to naturally suckling piglets. In fact, the piglets which were artificially fed with 100 mL of colostrum (porcine or goat) lost weight during the first 12 h, while the piglets of treatment C gained. This weight loss in artificially fed pigs suggests that 100 mL of colostrum might have been too low to meet the special nutrient needs of these animals. However, it is important to note that piglets fed with GC showed the same pattern of weight loss as those fed PC.

This low growth performance due to a low colostrum intake was compensated when the piglets were returned to a newly farrowing sow and weight gain during the rest of the suckling period was similar in both treatments. In addition, the body weight of artificially fed piglets at 20 days was comparable to that of the group C piglets. These results agree with the observations of Moreira et al. [26], who showed that performance during the first days of life may not have a direct effect on the weaning weight because of the many factors that influence performance in pigs.

Furthermore, the number of dead piglets was similar in all treatments, while no diarrhea or symptoms of intolerance were observed at any time. This is similar to the conclusions of Drew and Owen [10], who, using bovine IgG in newborn piglets, also failed to observe diarrhea at any time during the 28 days of their experiment.

Regarding IgG absorption, at 12 h, goat IgG appeared at very low levels in the piglet serum fed with C and PC treatments, probably due to cross-reactivity of the test. However, the concentration of IgG in piglet serum fed with GC was higher (8.11 mg/mL), while in these piglets, the level of porcine IgG was extremely low (1.41 mg/mL) compared with the rest of piglets (26.06 mg/mL and 66.24 mg/mL for piglets fed with PC and C treatments, respectively). Because the piglets fed with GC only received goat colostrum during the first 12 h after birth, the low concentration of porcine IgG in these piglets could be due to the same cross-reactivity or due to a minimum development of natural IgG during pig ontogeny [27]. The appearance of goat IgG levels in piglet serum are the first results that have shown that the piglets were able to absorb IgG from goat whole colostrum. Previous studies have shown that porcine and bovine IgG can be absorbed by piglets in the first hours after birth [5,8,11,28], but goat colostrum has not previously been used. Despite the tolerance shown by the piglets, IgG levels in piglets fed GC was lower than in piglets fed PC. This may be due to several reasons: on the one hand, the IgG concentration in porcine colostrum was higher than goat colostrum (87.47 *vs.* 41.60 mg/mL, respectively). Taking into account that serum concentrations of IgG in neonatal piglets are correlated with IgG intake [1,2] and, in this study, piglets fed PC and GC ingested the same amount of colostrum in the first 12 h after birth, it seems that the concentration of IgG colostrum played a decisive role. 

On the other hand, the absorption of colostral immunoglobulins is driven by both colostrum quality and the efficiency of absorption [23]. In this sense, the AEA of goat IgG was 20.9% and that of porcine IgG 26.3%. These results are comparable to those obtained by Bikker et al. [8] and Campbell et al. [9] for IgG obtained from sow colostrum and to those of other researchers when they used different sources of IgG in colostrum-deprived piglets. Jensen et al. [5] observed that newborn pigs fed cow colostrum showed a considerable uptake of macromolecules, including IgG, during the first 12 h after birth although in the presence of porcine IgG the piglets preferentially absorbed pig IgG relative to bovine IgG. In our study, at 12 h, goat IgG was not accompanied by porcine IgG, so these and previous results suggest that goat colostrum can be considered as a new alternative to sow colostrum for future studies.

Piglets fed GC that returned to a farrowing sow were able to absorb porcine IgG since intestinal macromolecular closure is well developed in most piglets by 18 h but intestinal closure is completed at 24–36 h [29,30]. Hence, we observed an increase in porcine IgG at 10 d, although its content was lower than in the other treatments. Fraser and Rhusel [31] concluded that the colostrum ingestion capacity of piglets is not homogeneous and is very high during the first 2 h of life. For this reason, piglets fed GC during the first 12 h were probably unable to ingest the same amount of sow colostrum and, therefore, of IgG, as those of the other treatments.

Serum level of porcine IgG in piglets of the C treatment was higher than the levels previously reported in naturally suckled piglets [8,11,32]. The serum level of IgG in piglets is related to several factors, particularly the content of Ig ingested [2]. In our study, while IgG in goat colostrum was in line with previously reported results [33,34], IgG concentration in sow colostrum was higher than in the above studies. For example, Bikker et al. [8] supplied sow colostrum with a content of 68.2 mg/mL of IgG, and Gomez et al. [11] used sow colostrum with 55.2 mg/mL, while colostrum used in this study contained 87.47 mg/mL. The higher serum porcine IgG concentration in C group compared with PC piglets could be indicative of the higher colostrum intake of C piglets since piglets fed PC ingested 100 mL during the first 12 h, while those of the C treatment probably ingested more. This would explain the growth results observed during the first 12 h of piglets fed with PC.

Therefore, goat colostrum can be considered a promising alternative as a new fed supplement or artificial rearing of newborn piglets. However, some researches have shown that colostrum-deprived neonatal pigs that were artificially reared with bovine colostrum during the first 2 d of life showed an 80% survival rate [11]. According to these data, future studies extending feeding time beyond 12 h with goat colostrum are necessary.

In this study, we used medium weight piglets, whereas low weight piglets are the most likely to die and most in need of supplementation after birth because they generally have a lower colostrum intake [29]. Hence, supplementation with goat colostrum in such piglets should also be investigated. 

## 5. Conclusions

In conclusion, this study demonstrates that neonatal piglets are capable of absorbing IgG from goat colostrum during the first 12 h after birth. In addition, goat colostrum is well tolerated by piglets during the first hours. To our knowledge, this is the first study showing the effects of goat colostrum in newborn piglets, but further research is necessary to explore the possibility of using this source, alone or in a mixture, as a supplement for the artificial rearing of newborn piglets.

## Figures and Tables

**Table 1:** Chemical composition and immunoglobin G (IgG) concentration of goat and sow colostrum fed to newborn piglets (as-fed basis).

Item	Goat Colostrum	Sow Colostrum
Dry matter (g/kg)	222.0	274.0
Crude protein (g/kg)	102.7	182.0
Fat (g/kg)	83.70	43.00
Lactose (g/kg)	31.20	30.80
IgG (mg/mL)	41.60	87.47

**Table 2:** Body weight, weight gain, and rectal temperature of piglets across the different experimental treatments.

	Treatment ^1^			SEM	*p*-Value
Item	C	PC	GC		
Body weight (g)					
Initial (0 h)	1330	1323	1302	30.42	0.931
Final (20 d)	4531	4217	4543	132.9	0.534
Weight gain (g) ^2^					
0 h–12 h	84.6 a	−53.5 b	−52.6 b	7.957	<0.001
12 h–24 h	90.67	111.0	112.2	11.30	0.639
24 h–10 d	1674	1693	1656	70.11	0.979
0 h–20 d	3175	2871	3248	134.1	0.495
Temperature (°C)					
0 h	37.5	37.9	37.6	0.165	0.542
24 h	38.0	37.7	37.9	0.103	0.362

**^1^** C: Control group, where piglets were kept with their own sows and allowed to suckle pig colostrum as normal. PC: Piglets were removed from the sows after birth and received porcine colostrum only for the first 12 h of life. GC: Piglets were removed from the sows after birth and received goat colostrum only for the first 12 h of life. The analysis was performed as a univariate analysis by General Linear Model and predictor variable was the treatment. ^2^: weight at 0 h corresponded to the initial birth weight. a, b: Means in the same row bearing the same letter are not significantly different (*p* > 0.05).

**Table 3:** Concentration of goat and pig IgG of piglets’ serum across the different experimental treatments.

	Treatment ^1^			SEM	*p*-Value
Item	C	PC	GC		
Goat IgG ( mg/mL)					
12 h	0.51 b	0.40 b	8.11 a	1.398	0.014
10 d	<0.01	<0.01	0.13	0.023	0.116
Pig IgG (mg/mL)					
12 h	66.24 a	26.06 b	1.41 c	2.338	<0.001
10 d	35.39 a	23.09 b	10.00 c	1.665	<0.001
20 d	16.99	19.88	11.47	1.553	0.124

^1^ C: Control group where piglets were kept with their own sows and allowed to suckle pig colostrum as normal. PC: Piglets were removed from the sows after birth and received porcine colostrum only for the first 12 h of life. GC: Piglets were removed from the sows after birth and received goat colostrum only for the first 12 h of life. a–c: Means in the same row bearing the same letter are not significantly different (*p* > 0.05).
